# FYN expression potentiates FLT3-ITD induced STAT5 signaling in acute myeloid leukemia

**DOI:** 10.18632/oncotarget.7128

**Published:** 2016-02-02

**Authors:** Rohit A. Chougule, Julhash U. Kazi, Lars Rönnstrand

**Affiliations:** ^1^ Division of Translational Cancer Research, and Lund Stem Cell Center, Department of Laboratory Medicine, Lund University, Lund, Sweden

**Keywords:** FYN, FLT3, FLT3-ITD, STAT5, acute myeloid leukemia

## Abstract

FYN is a non-receptor tyrosine kinase belonging to the SRC family of kinases, which are frequently over-expressed in human cancers, and play key roles in cancer biology. SRC has long been recognized as an important oncogene, but little attention has been given to its other family members. In this report, we have studied the role of FYN in FLT3 signaling in respect to acute myeloid leukemia (AML). We observed that FYN displays a strong association with wild-type FLT3 as well as oncogenic FLT3-ITD and is dependent on the kinase activity of FLT3 and the SH2 domain of FYN. We identified multiple FYN binding sites in FLT3, which partially overlapped with SRC binding sites. To understand the role of FYN in FLT3 signaling, we generated FYN overexpressing cells. We observed that expression of FYN resulted in slightly enhanced phosphorylation of AKT, ERK1/2 and p38 in response to ligand stimulation. Furthermore, FYN expression led to a slight increase in FLT3-ITD-dependent cell proliferation, but potent enhancement of STAT5 phosphorylation as well as colony formation. We also observed that FYN expression is deregulated in AML patient samples and that higher expression of FYN, in combination with FLT3-ITD mutation, resulted in enrichment of the STAT5 signaling pathway and correlated with poor prognosis in AML. Taken together our data suggest that FYN cooperates with oncogenic FLT3-ITD in cellular transformation by selective activation of the STAT5 pathway. Therefore, inhibition of FYN, in combination with FLT3 inhibition, will most likely be beneficial for this group of AML patients.

## INTRODUCTION

Expression of receptor tyrosine kinases has been shown to be deregulated in many cancers. Deregulation can be due to overexpression or through oncogenic mutations. A member of the PDGFR-family receptor tyrosine kinase (also called type III receptor tyrosine kinases), FLT3, has been shown to be deregulated in acute myeloid leukemia (AML) and in a small portion of acute lymphoblastic leukemia [1, 2]. FLT3 was found to be mutated in as high as 35% of AML patients. The most common mutation in FLT3 is the internal tandem duplication (ITD) and other mutations include point mutations in the kinase domain.

The downstream signaling of FLT3 is tightly controlled by FLT3 associating proteins. Activation of FLT3 is mediated through ligand binding and dimerization followed by phosphorylation of several tyrosine residues in the intracellular domain. Phosphorylation of tyrosine residues creates docking sites for the SH2 domain-containing proteins that further propagate or terminate signaling. For example association of non-receptor tyrosine kinases SRC and SYK, adaptor proteins GRB10 and SLAP resulted in enhancement of FLT3 signaling [3–5]. In contrast, the association of other SH2 domain containing proteins such as CBL ubiquitin ligases, SOCS2 and SOCS6 adaptor proteins, LNK and the non-receptor tyrosine kinase CSK negatively regulates FLT3 signaling [6–9]. Therefore, understanding the role of FLT3 binding proteins is of importance to understand FLT3 mediated pathogenesis in AML.

The proto-oncogene FYN is a member of the SRC family of protein tyrosine kinases (SFKs). The SFKs includes 11 non-receptor protein tyrosine kinases [10]. All family members share a similar structure and arrangement of functional domains. The important features of this family proteins are presence of a kinase domain (initially was known as SRC homology 1 or SH1 domain), a SRC homology 2 (SH2) domain and a SRC homology 3 (SH3) domain. The presence of the SH2 domain facilitates association with tyrosine phosphorylated proteins, while the SH3 domain is involved in association with proteins containing proline-rich (PXXP) motifs. Therefore, SFKs associate with a wide range of proteins including receptors, adaptors and scaffolding proteins. FYN is involved in distinct cellular signaling modulating signals from Eph receptors [11], the T-cell receptor [12], and the epidermal growth factor receptor (EGFR) [13]. FYN has also been implicated in cancer [14]. A report suggests that the expression of FYN predicts the long-term survival of MYCN negative neuroblastoma cells [15]. In chronic myeloid leukemia (CML) FYN expression was upregulated and depletion of FYN was associated with increased sensitivity to imatinib as well as decreased cell growth and colony formation [16]. Furthermore activation of FYN resulted in resistance to the BCR-ABL inhibitor imatinib [17]. These findings suggest that FYN plays an important role in cancer and can be a potential drug target for cancer therapy.

In this report we describe a novel role of FYN in FLT3 signaling. We show that FYN interacts with wild-type and oncogenic FLT3 through its SH2 domain in a FLT3 kinase-dependent manner. This association has no effect on FLT3 stability but overexpression of FYN in FLT3 expressing Ba/F3 cells slightly enhanced mitogenic signaling. Moreover, FYN expression significantly enhanced FLT3-ITD-mediated STAT5 phosphorylation as well as colony formation in semi-solid medium.

## RESULTS

### Elevated FYN expression correlates with poor patient survival in AML

**Figure 1:**
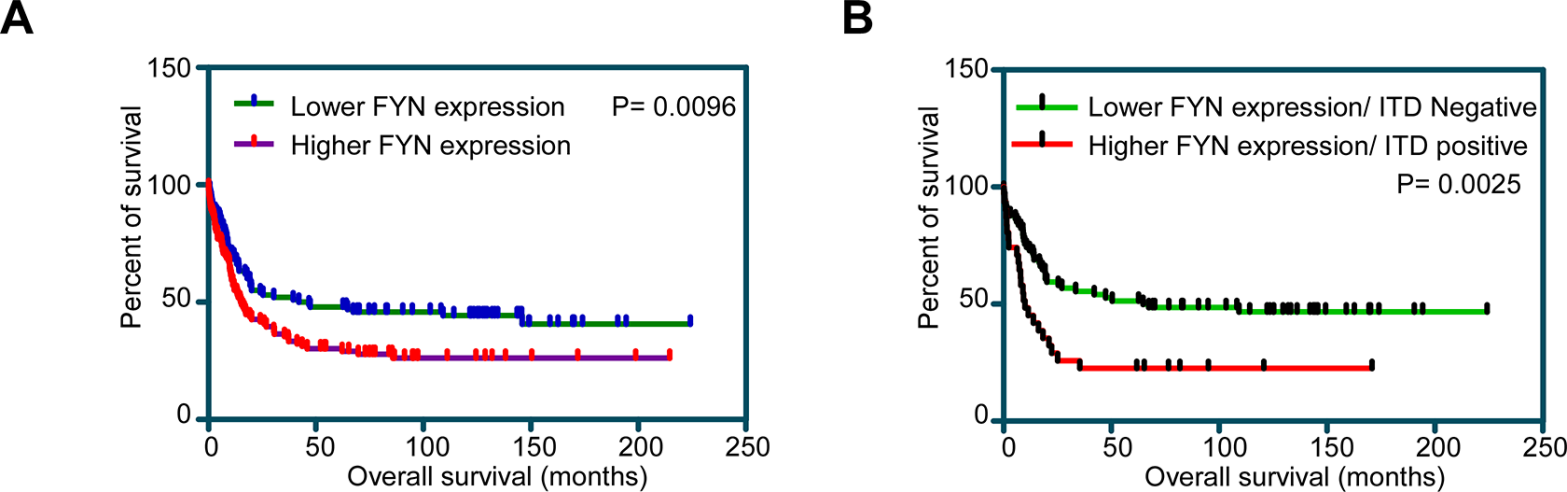
Overall survival of AML patients with higher and lower FYN expression: Z-score of mRNA expression from data set GSE14468 was used to divide higher (*n* = 40) and lower (*n* = 40) FYN expressing patients (**A**) Overall survival of AML patients with higher or lower FYN expression independent of FLT3-ITD expression. (**B**) Overall survival of AML patients in lower FYN expression and FLT3-ITD negative versus higher FYN expression and FLT3 positive.

To understand the role of SFKs in AML we analyzed the expression patterns of SFKs using mRNA expression data from AML patient samples. Among the eleven SFKs, FGR, FYN, HCK, LCK, LYN and YES1 displayed differential expression patterns (Figure S1A). Since these six SFKs displayed differential expression patterns in AML patients, we then analyzed whether differential expression of SFKs has a prognostic significance. We divided patients into two groups depending on whether they had high or low expression of individual SFKs. Z-score of mRNA expression were calculated for individual SFKs and 40 patients with highest Z-score and 40 patients with lowest Z-score were used for analysis. We observed that only high expression of FYN (Figure 1A) but not FGR (Figure S1B), LYN (Figure S1C), HCK (Figure S1D), LCK (Figure S1E) and YES1 (Figure S1F), correlated with poor patient survival (*P* = 0.0096). FLT3-ITD is the most commonly mutated gene in AML and correlates with poor prognosis. We observed that patient displaying both high FYN expression and FLT3-ITD mutations further showed poor survival (*P* = 0.0026) in comparison to patient with low FYN expression (Figure 1B). Therefore, we suggest that FYN may play a role in AML patients carrying FLT3-ITD.

### FYN associates with tyrosine phosphorylated FLT3

**Figure 2:**
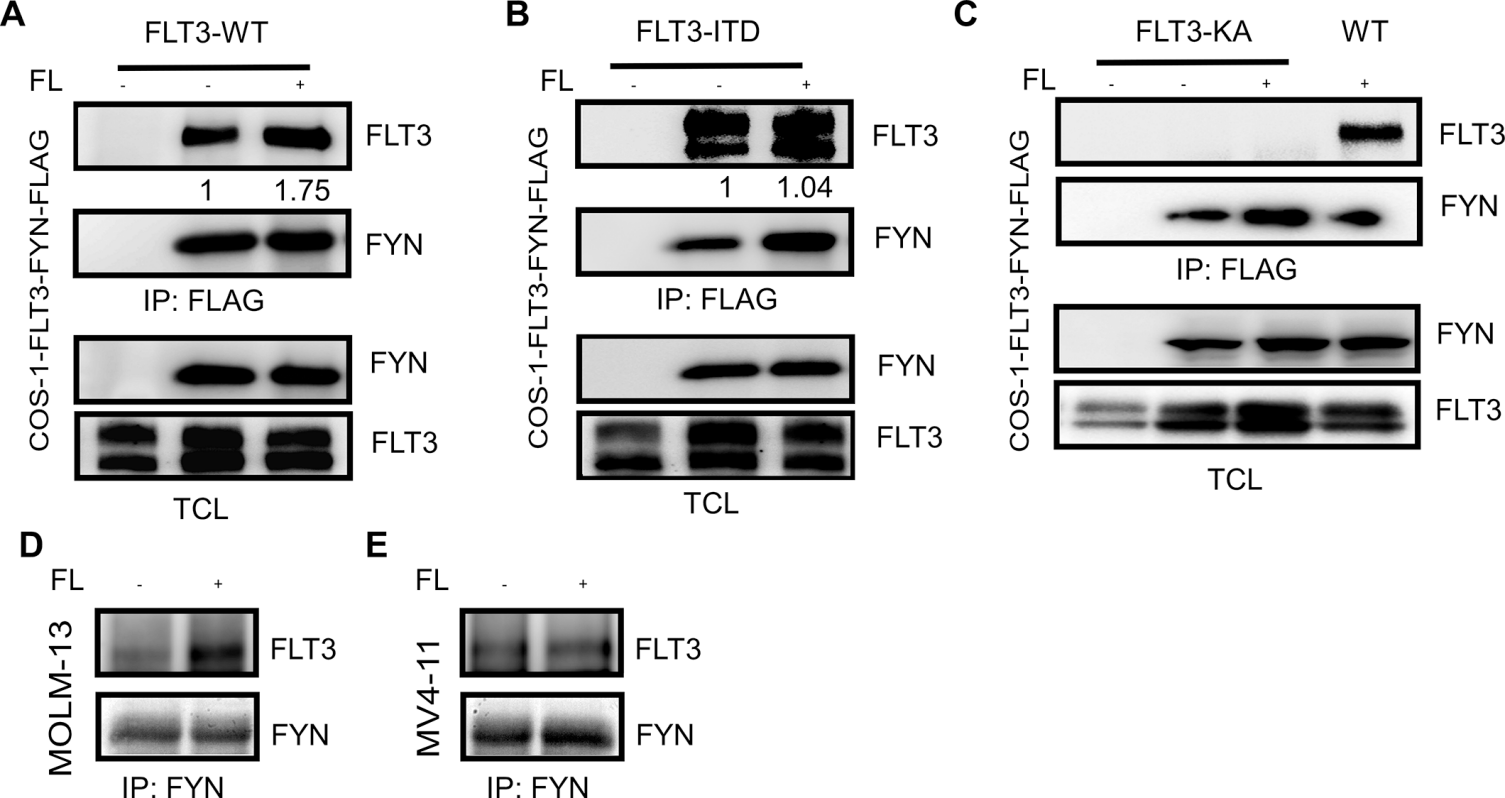
FLT3 associates with FYN in a phosphorylation-dependent manner COS1 cells were transfected with FLAG-tag FYN along with plasmids expressing FLT3-WT (**A**), FLT3-ITD (**B**) or FLT3-K644A. After 5 minutes of stimulation with 100 ng/ml FL, cells were lysed. Cell lysates were subjected to the anti-FLAG antibody immunoprecipitation followed by SDS-PAGE separation and western blotting analysis.

Among the SRC family kinases (SFKs), SRC [18], HCK [19] and LYN [20] have been shown to interact with FLT3 and play important roles in FLT3 maturation and signaling [21–23]. The role of SRC in FLT3-ITD-induced downstream signaling remains debated. While one report suggested that SRC is not involved in FLT3-ITD-induced STAT5 activation [24], another report suggested the involvement of SRC in STAT5 activation [18]. To understand the involvement of FYN in FLT3 signaling, we initially checked whether FYN associates with FLT3. We co-expressed FLAG-tagged FYN with wild-type FLT3 in COS-1 cells. We observed a strong association between FYN and wild-type FLT3 which was enhanced by FLT3 ligand (FL) stimulation (Figure 2A). It was not completely unexpected that FYN associates wild-type FLT3 in the absence of ligand stimulation in COS-1 cells, as overexpression of wild-type FLT3 results in ligand-independent activation of FLT3 (data not shown). Furthermore, FYN associated with FLT3-ITD in a ligand-independent manner (Figure 2B). Even though, overexpression of FLT3 in COS-1 cells resulted in ligand-independent-activation of FLT3, it was difficult to conclude that the interaction between FYN and FLT3 was mediated through FLT3 tyrosine phosphorylation, although we observed an increase in FLT3 co-immunoprecipitation in ligand stimulated cells (Figure 2A). To resolve this question, we used a kinase-dead mutant of FLT3 [25]. As we observed that wild-type FLT3 associates with FYN, the FLT3-KA mutant was unable to interact with FYN (Figure 2C). Furthermore, FYN and FLT3 association was detected in AML cell lines MOLM-13 (Figure 2D) and MV4-11 (Figure 2E). Therefore, our data suggest that the FLT3 kinase activity is essential for the interaction with FYN. In other words, FYN associates with FLT3 through phosphorylated tyrosine residues.

### FYN associates with the FLT3 pY591, pY599 and pY955 residues through its SH2 domain

**Figure 3:**
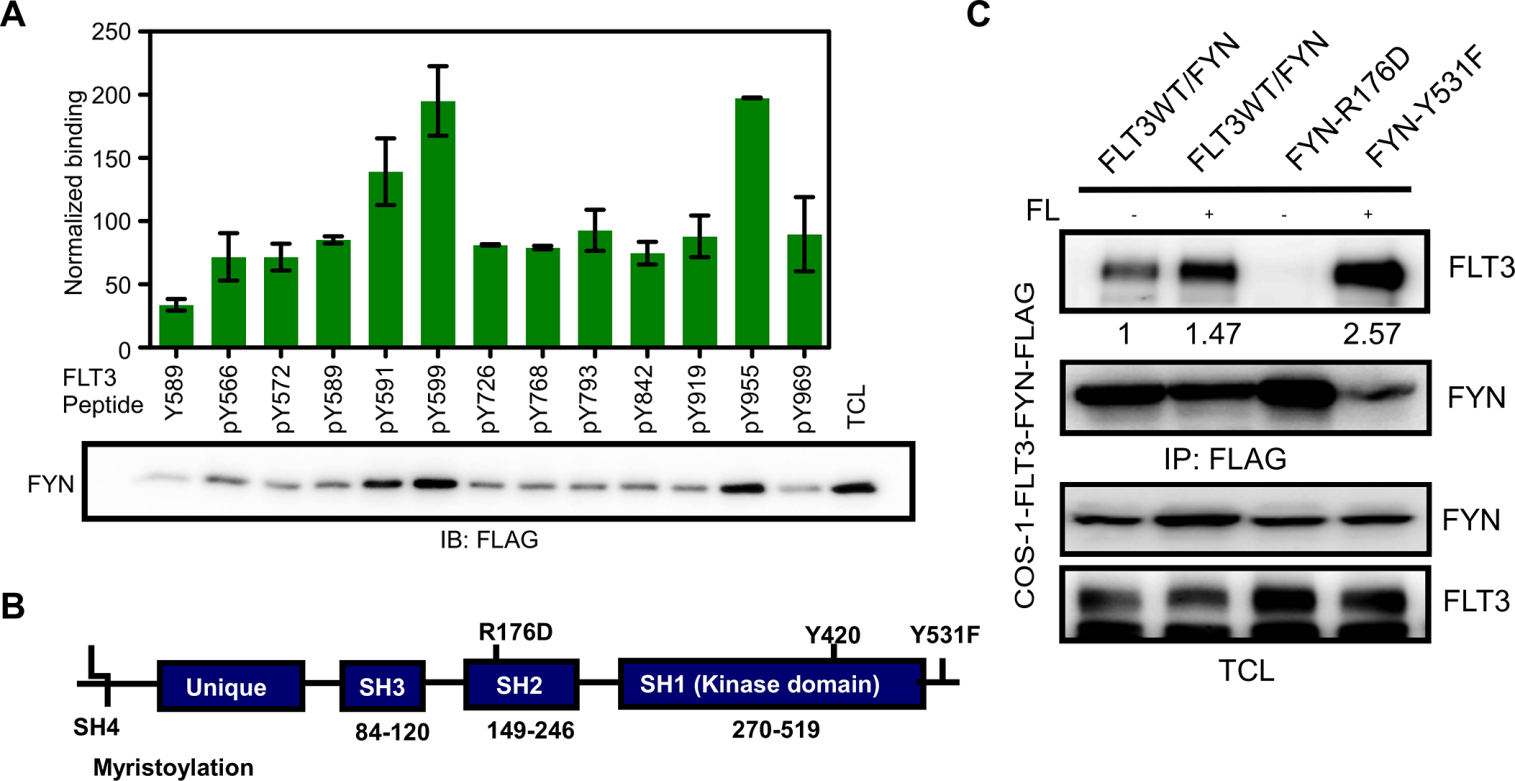
FYN associates with FLT3 through the FYN SH2 domain (**A**) Synthetic phospho-peptides corresponding to known and predicted FLT3 tyrosine phosphorylation sites were used to pull down FYN from cells lysates of COS1 cells transfected with FYN. (**B**) Schematic presentation of FYN. (**C**) COS1 cells were transfected with plasmids expressing FLT3-wild-type and FYN wild-type or mutants. After 5 minutes of stimulation with 100 ng/ml of FL, cells were lysed. Cell lysates were subjected to immunoprecipitation with an anti-FLAG antibody followed by SDS-PAGE separation and western blotting analysis.

We then aimed at identifying the binding sites in FLT3. Phosphorylation of FLT3 on tyrosine residues occurs at multiple sites in the intracellular domain. We used tyrosine-phosphorylated synthetic peptides corresponding to known or predicted tyrosine phosphorylation sites in FLT3 to identify candidate FYN interaction sites. We observed that FYN associates with FLT3 through binding to the FLT3-pY591, FLT3-pY599 and FLT3-pY955 residues (Figure 3A). These residues are partially identical to the SRC binding sites in FLT3 [21]. FYN has three well-characterized domains including SRC homology 1 (SH1) domain (i. e. the kinase domain), SH2 domain and SH3 domain (Figure 3B). The SH2 domain is well-known to interact with phosphorylated tyrosine residues. To test whether the FYN SH2 domain is involved in association with tyrosine phosphorylated FLT3, we generated a FYN-R176D mutant. The arginine 176 residue in the SH2 domain of FYN is known to be involved in interaction with phosphorylated tyrosine residues. We observed that association between FLT3 and the FYN-R176D mutant was completely abolished (Figure 3C), suggesting that FLT3 associates with FYN through the FYN SH2 domain. Furthermore, a constitutively active FYN mutant, FYN-Y531F, displayed slightly increased association with FLT3.

### The FLT3-FYN interaction is required for FLT3-mediated tyrosine phosphorylation of FYN

**Figure 4:**
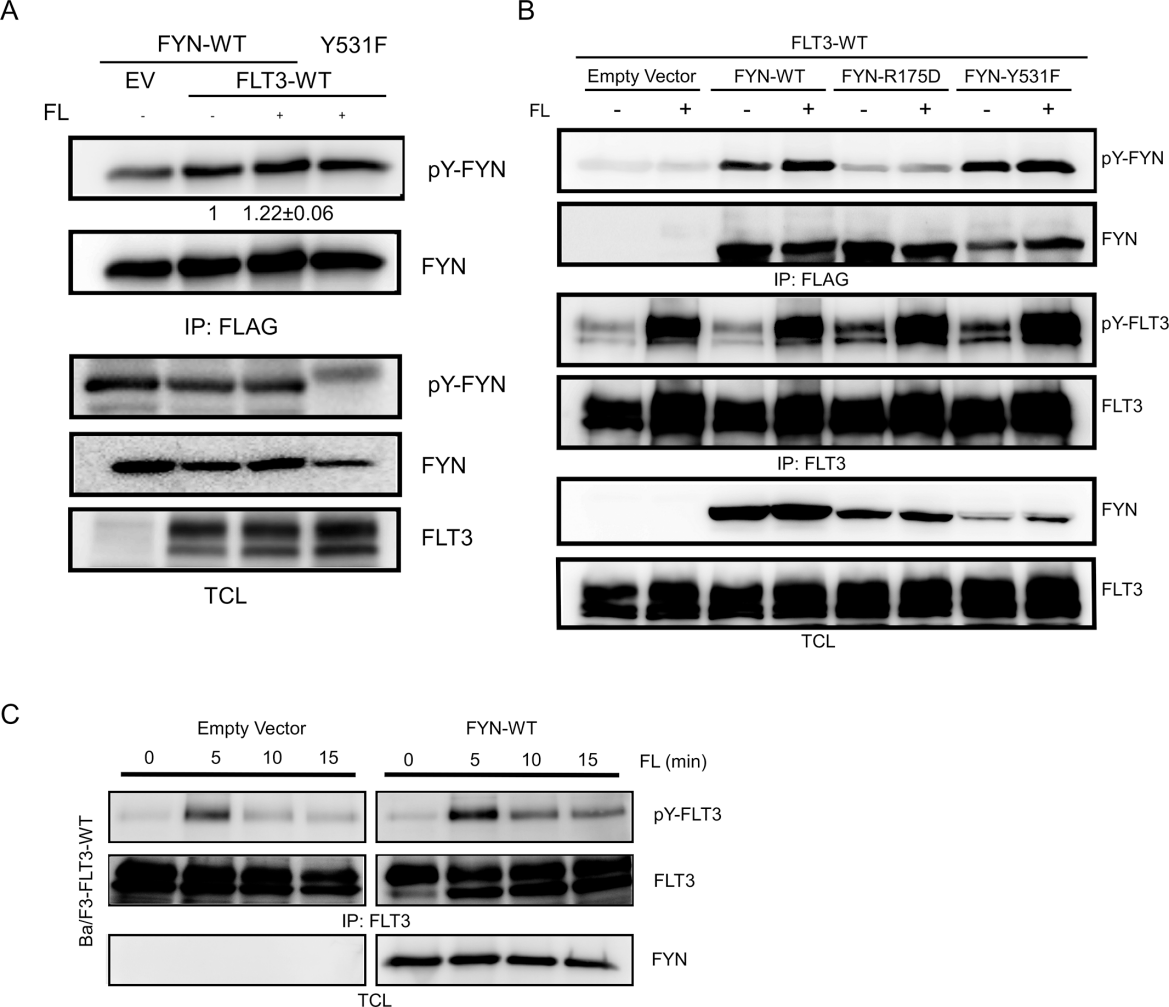
FYN phosphorylates FLT3 on tyrosine residues (**A**–**B**) COS1 cells were transfected with plasmids expressing FLT3 wild-type and FYN wild-type or mutants. Cells were stimulated with 100 ng/ml FL for 5 minutes followed by lysis. Cell lysates were subjected to immunoprecipitation with an anti-FLAG or anti-FLT3 antibody followed by SDS-PAGE separation and western blotting analysis. (**C**) Ba/F3 cells stably expressing FYN and FLT3 were stimulated with FL for different periods of time. Cells were lysed and subjected to immunoprecipitation with an anti-FLT3 antibody.

FYN is phosphorylated on several tyrosine residues. We observed that wild-type FYN remains tyrosine phosphorylated in COS-1 cells and ligand stimulation slightly increased tyrosine phosphorylation of FYN (Figure 4A). However, tyrosine phosphorylation of the SH2 domain mutant FYN-R176D was much lower (Figure 4B) suggesting that the FLT3-FYN interaction is required for tyrosine phosphorylation of FYN. Moreover, cells expressing FYN displayed slightly higher tyrosine phosphorylation of FLT3 in comparison to cells transfected with empty control vector (Figure 4C).

### FYN does not influence FLT3 stability

**Figure 5:**
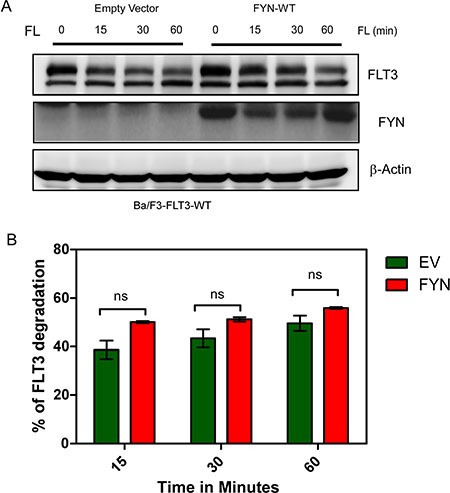
FYN expression does not affect FLT3-ITD stability (**A**) Ba/F3 cells were washed three times with RPMI-1640 to remove IL3. Cells were then incubated with cycloheximide for 30 minutes before stimulation with FL. (**B**) Band intensity of multiple blots were quantified by MultiGauge, ns, not significant.

Although we observed that FYN expression increased FLT3 tyrosine phosphorylation, we did not see any considerable increase in ubiquitylation of FLT3 in FYN transfected cells (data not shown). Furthermore, time-dependent stimulation with FL in the presence of cycloheximide, an inhibitor of protein synthesis, did not result in any significant difference in FLT3 degradation (Figure 5A and 5B).

### FYN expression slightly increases FL-induced AKT, ERK and p38 phosphorylation in Ba/F3-FLT3-WT cells

**Figure 6:**
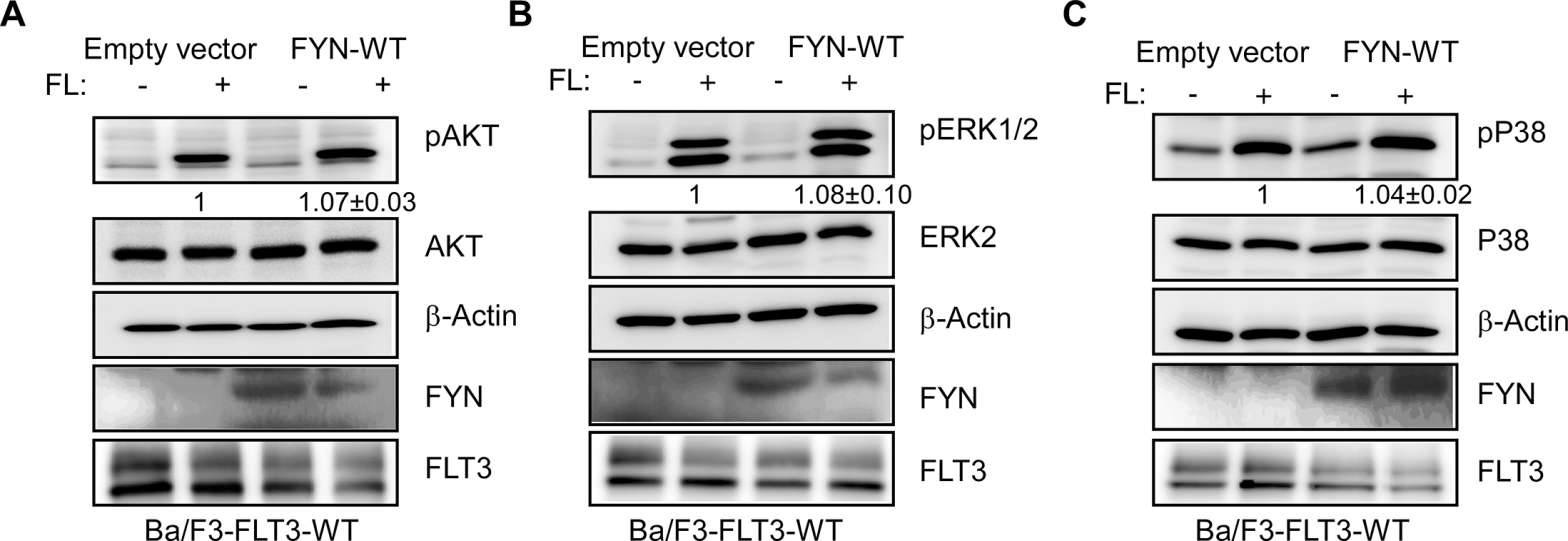
FYN expression slightly increased AKT, ERK1/2 and p38 phosphorylation Ba/F3/FLT3-WT cells stably transfected with FYN or empty vector were washed three times with RPMI-1640 to remove IL3. (**A**–**C**) Cells were stimulated with a ligand for 5 minutes before lysis. Total cell lysates were used for SDS-PAGE and western blotting analysis with AKT (A), ERK (B) and p38 (C) antibodies.

To understand the role of FYN in FLT3 signaling we used Ba/F3-FLT3-WT/empty vector and Ba/F3-FLT3-WT/FYN cell lines that overexpress FYN. Activation of wild-type FLT3 resulted in activation of the AKT, ERK and p38 signaling pathways. We observed that FYN expression slightly increased FL-induced AKT phosphorylation (Figure 6A) but did not alter FL-induced ERK 1/2 (Figure 6B) or p38 (Figure 6C) phosphorylation.

### FYN expression enhances FLT3-ITD induced STAT5 signaling

**Figure 7:**
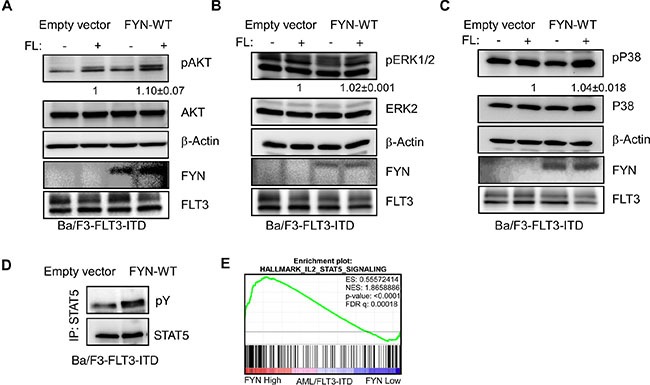
FYN expression significantly increases FLT3-ITD-induced STAT5 phosphorylation: Ba/F3/FLT3-ITD cells stably transfected with FYN or empty vector were washed three times with RPMI-1640 to remove IL3 (**A**–**C**) Cells were stimulated with ligand for 5 minutes before lysis. Total cell lysates were used for SDS-PAGE and western blotting analysis with AKT (A), ERK (B) and p38 (C) antibodies. (**D**) Cell lysates from unstimulated cells were immunoprecipitated with an anti-STAT5 antibody followed by western blotting analysis. (E) GSEA showed enrichment of the IL2/STAT5 pathways in AML patients expressing higher levels of FYN. Z-score of FYN expression from data set GSE14468 was used to divide higher (*n* = 40) and lower (*n* = 40) FYN expressing patients.

Wild-type FLT3 and FLT3-ITD differ in their ability to activate downstream signaling. While wild-type FLT3 activates AKT, ERK and p38 signaling, FLT3-ITD activates in addition to AKT, ERK and p38 signaling also STAT5 signaling. Similar to what we saw with wild-type FLT3, we observed a slight increase in AKT phosphorylation in Ba/F3-FLT3-ITD/FYN cells (Figure 7A) but not in ERK 1/2 Figure 7B) or p38 (Figure 7C) phosphorylation. However, Ba/F3-FLT3-ITD cells expressing FYN displayed significantly higher STAT5 phosphorylation compared to empty vector (Figure 7D). Furthermore, gene set enrichment analysis (GSEA) using AML patient data showed that the STAT5 pathway is enriched in a group of patients with higher FYN expression (Figure 7E). Seventy-two signature proteins were significantly upregulated in higher FYN expressing patient group. Furthermore, using the panther classification system (http://pantherdb.org) many of those genes are found to be involved in regulation of cell survival.

### FYN expression did not alter FLT3-ITD-induced cell proliferation and viability but increased the ability to form colonies

**Figure 8:**
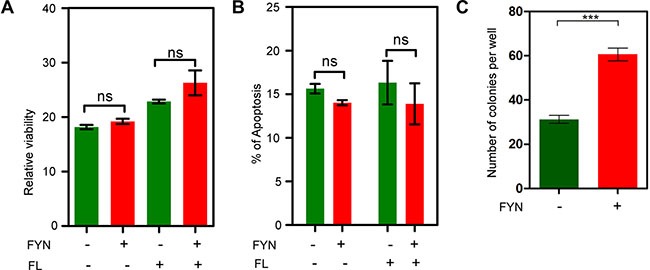
FYN expression significantly contributes to colony formation Ba/F3/FLT3-ITD cells stably transfected with FYN or empty vector were washed three times with RPMI-1640 to remove IL3. (**A**) FLT3-ITD dependent cell proliferation in presence or absence of FYN expression was measured after 48 hours using PrestoBlue cell viability assay. (**B**) Apoptosis response upon cytokine depletion was measured using Annexin-V and 7-AAD kit. (**C**) Around 1000 cells were seeded in semi-solid medium followed by counting colonies after seven days.

Since we observed that FYN expression correlated with poor prognosis in AML patients carrying FLT3-ITD mutations and since FYN associates with both FLT3-WT and FLT3-ITD, we hypothesized that FYN may play a role FLT3-induced biological outcomes. We generated Ba/F3 cell lines expressing FLT3-WT or FLT3-ITD along with FYN or empty control vector. FYN expression slightly potentiated FLT3-ITD-induced cell proliferation (Figure 8A) and slightly reduced apoptosis in the absence of cytokines (Figure 8B). However, the effect of FYN on cell proliferation and apoptosis was not statistically significant. We also performed colony formation assay in semi-solid medium. FYN expression dramatically increased the number of colonies in semi-solid culture (Figure 8C). In addition, using a STAT5-specific inhibitor we observed that anchorage-independent growth was completely abolished in FYN expressing cells (data not shown) suggesting that STAT5 is a target of FLT3-FYN signaling. Therefore, we suggest that FYN cooperates with FLT3-ITD in colony formation but not cell proliferation or survival signaling.

## DISCUSSION

In order to further understand FLT3-ITD induced AML pathogenesis, we defined the role of FYN in FLT3-ITD dependent AML. In a screen using mRNA data from AML patient samples we observed that among the 11 SFKs, FYN expression correlated with AML patient survival. Therefore, we aimed to understand the role of FYN in FLT3-ITD - dependent AML. We showed that FYN is involved in FLT3-ITD - induced transformation. FYN has previously been found to associate with other type III receptor tyrosine kinases such as KIT and PDGFRβ. FYN associates with KIT through the major SFKs binding site Y568 in KIT [26]. A specific role for FYN in KIT signaling was not defined in that study but rather the common roles of SFKs by mutating SFK binding sites, thus affecting all SFKs expressed in the cells studied. Association of FYN with PDGFRβ potentiates receptor tyrosine phosphorylation [27]. Furthermore, ligand-stimulation of PDGFRβ also induced tyrosine phosphorylation of FYN [28]. We also observed that FYN expression increased ligand-induced FLT3 phosphorylation, while FLT3 activation also increased FYN phosphorylation suggesting that activation of FLT3 and FYN are partially dependent on each other.

Due to high degree of structural similarity and abundant expression of many family members in most tissues, it still remains a challenge to study the roles of individual SFKs. We still do not have any highly specific SFK inhibitors. The major FYN interaction residues in FLT3, pY591 and pY599, are also well-known SRC binding sites [18, 21]. Additionally, FYN displays a novel binding site, pY955, which has not yet been described as a SFK binding site. The pY955 site in FLT3 is involved in interaction with the adaptor protein Grb2, which associates with FLT3 through pY768, pY955 and pY969 [29]. Therefore, it is likely that FYN has additional roles in FLT3 signaling that other SFKs don't.

The exact mechanism by which FYN regulates STAT5 phosphorylation is not known. We have previously demonstrated that the scaffolding protein GAB2 is involved in regulation of STAT5 phosphorylation downstream of FLT3-ITD [29]. Phosphorylation of GAB2 on key tyrosine residues creates binding sites for a number of signal transduction molecules, including PI3-kinase and SHP2, which relay the signal into the cell (for review, see [30]. Several tyrosine kinases have been suggested to induce phosphorylation of GAB2 and mediate activation of downstream signaling pathways. One of these kinases is FYN. In FcεRI-mediated mast cell granulation, the FYN/GAB2/RHOA pathway plays an important role [31]. Other tyrosine kinases that have been implicated in the regulation of STAT5 phosphorylation downstream of FLT3-ITD include the closely related FER and FES [32]. siRNA-mediated knockdown of either FER or FES strongly reduced STAT5 phosphorylation and partially suppressed phosphorylation of SRC family kinases in FLT3-ITD expressing cells, suggesting that some but not all SRC family kinases are involved in regulation of STAT5 phosphorylation.

The minimal differences in phosphorylation of AKT, ERK1/2 and p38 seen in FYN overexpressing cells is probably due to the fact that FLT3 downstream signaling is already saturated by endogenous expression of the predominant SFKs expressed in Ba/F3 cells, SRC and LYN, and probably the pY591 and pY599 sites are the major phosphorylation sites in FLT3 signaling that are required for activation of AKT, ERK1/2 and p38. FYN plays a unique role in STAT5 signaling since overexpression of FYN selectively elevated STAT5 phosphorylation as well as colony formation. In addition to the cell lines studied, we observed an enrichment of the STAT5 pathway in AML patients with higher FYN expression. Furthermore, AML patient carrying the FLT3-ITD mutation combined with higher FYN expression displayed relatively poor prognosis. Collectively we suggest that FYN by activating STAT5 signaling plays an important role in FLT3-ITD-mediated transformation in AML and therefore a combinatorial therapy targeting FYN in addition to FLT3-ITD would likely be beneficial for this group of patients.

## MATERIALS AND METHODS

### Data from AML patient samples

Data from AML patient samples were collected from the data set GSE14468 [33]. This data set contains mRNA expression data from 524 AML patient samples. Corresponding patient survival data was kindly provided by Dr. Peter J. Valk at Erasmus University. Normalized data were downloaded from NCBI GEO (http://www.ncbi.nlm.nih.gov/geo/query/acc.cgi?acc=GSE14468).

### Cell culture

The murine hematopoietic cell line Ba/F3 was cultured in RPMI-1640 media (Hyclone, Thermo Scientific, Waltham, MA) supplemented with 10% heat-inactivated fetal bovine serum (Life Technologies, Carlsbad, CA), 1% penicillin and streptomycin and 10 ng/ml murine interleukin 3 (IL3) as previously described [34]. COS-1 cells were cultured in DMEM supplemented with 10% fetal bovine serum (Life Technologies, Carlsbad, CA) and 1% penicillin and streptomycin. Cells were grown in a humidified atmosphere containing 5% CO_2_ at 37°C.

### Plasmids, antibodies and inhibitors

The open reading frame (ORF) of human FYN (BC032496) was subcloned (BamHI-XhoI) into a modified pcDNA3.1 vector pcFLAG [8] expressing a full-length FYN protein with a C-terminal FLAG-tag. For retroviral transduction, the pMSCVneo-FYN-WT-Myc-FLAG plasmid was generated by ligating full-length human FYN into the SgfI-MluI site in the place of BEX1 gene from pMSCVneo-BEX1-WT-Myc-FLAG plasmid [35]. FYN-R176D and FYN-Y531F mutants were generated by site-directed mutagenesis [36]. Anti-FLT3, 4G10, Anti-phospho AKT, anti-phospho-ERK, Anti-phospho p38, anti-AKT, anti-ERK and anti-p38 antibodies were described elsewhere [37–39].

### Stable transfection of Ba/F3

To establish Ba/F3 cells stably expressing FLT3-WT and FLT3-ITD, EcoPack packaging cells were transfected with pMSCVpuro-FLT3-WT or pMSCVpuro-FLT3-ITD construct, and virus-containing supernatants were collected 72 h after transfection as described previously [40, 41]. Retroviral infection of Ba/F3 cells was followed by a 2-week selection in 1.2 μg/ml puromycin. FLT3 expression was confirmed by flow cytometry and western blotting. Ba/F3-FLT3-WT and Ba/F3-FLT3-ITD cells were then further infected with virus produced in EcoPack cells by transfecting with pMSCVneo-FYN-Myc-FLAG construct or empty vector. Three days after infection cells were selected with 0.8 mg/ml G-418 for 2 weeks, and FYN expression was verified by western blotting.

### Immunoprecipitation, western blotting and peptide fishing

After 100 ng/ml FL-stimulation cells were washed once with cold PBS and lysed using Triton X-100 based lysis buffer. Immunoprecipitation and western blotting were performed following the previously describe methods [42]. Phospho-peptide corresponding known and predicted tyrosine phosphorylation sites (sequence described in reference [38]) were immobilized to the ultralink beads and 1:1 slurry of beads was used to pull-down FYN from COS-1 cell lysates.

### Apoptosis, cell proliferation and colony formation assays

Apoptosis was measured using annexin V and 7-aminoactinomycin D (7-AAD) kit (BD biosciences) following the previously described method [43]. For the cell proliferation assay, cells were seeded in a 96-well plate and incubated for 48 hours. Cell viability was measured using PrestoBlue cell viability assay. For colony formation assays, cells were seeded in semi-solid methylcellulose medium (Stem Cell Technologies) and then cultured for seven days before counting colonies [44].

## SUPPLEMENTARY MATERIALS FIGURE


