# Supplementary material for: FYN expression potentiates FLT3-ITD induced STAT5 signaling in acute myeloid leukemia
Source: Oncotarget. 2016 Feb 2;7(9):9964–74. doi: 10.18632/oncotarget.7128 (PMC4891096; doi:10.18632/oncotarget.7128)
Supplement: Supplementary file 1 [file oncotarget-07-09964-s001.pdf]

FYN expression potentiates FLT3-ITD induced STAT5 signaling in acute myeloid leukemia

Supplementary Materials

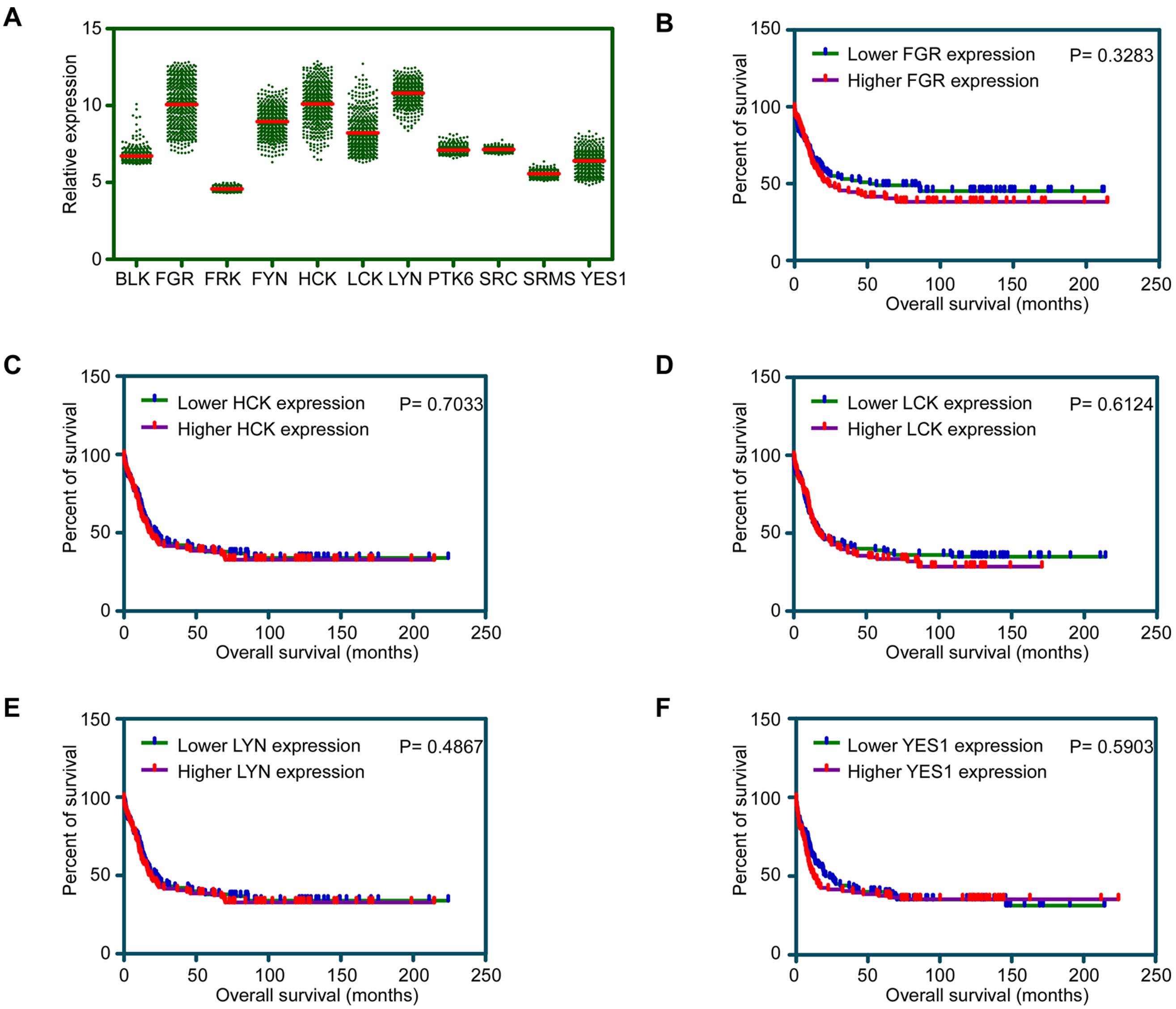

Supplementary Figure S1: Overall survival of AML patients with higher and lower FYN expression. Z-score of mRNA expression from data set GSE14468 was used to divide higher ($n = 40$) and lower ($n = 40$) FYN expressing patients. (**A**) Expression of SRC family proteins in AML. (**B**–**F**) Overall survival of AML patients with higher or lower FGR (B), HCK (C), LCK (D), LYN (E) and YES1 (F) expression independent of FLT3-ITD expression.
